# Response Surface Optimization of Sustained Release Metformin-Hydrochloride Matrix Tablets: Influence of Some Hydrophillic Polymers on the Release

**DOI:** 10.5402/2012/364261

**Published:** 2012-09-04

**Authors:** Amitava Roy, Kalpana Roy, Sarbani Roy, Jyotirmoy Deb, Amitava Ghosh, Kazi Asraf Ali

**Affiliations:** ^1^Department of Pharmaceutics, Himalayan Pharmacy Institute, East Sikkim, Majhitar, India; ^2^Department of computer Sciences, Dr. B.C. Roy Engineering College, Durgapur 713206, India; ^3^College of Pharmacy, Gupta College of Technological Sciences, Asansol, Burdwan 713301, India; ^4^Bengal College of Pharmaceutical Sciences and Research, Bidhannagar, Durgapur 713212, India; ^5^Department of Pharmaceutical Technology, Jadavpur University, Kolkata 700032, India

## Abstract

The aim of the present work was designed to develop a model-sustained release matrix tablet formulation for Metformin hydrochloride using wet granulation technique. In the present study the formulation design was employed to statistically optimize different parameters of Metformin hydrochloride tablets at different drug-to-polymer ratios employing polymers Hydroxypropyl methylcellulose of two grades K4M and K100M as two independent variables whereas the dependent variables studied were X_60_, X_120_, T_50_, T_90_, *n*, and *b* values obtained from dissolution kinetics data. The *in vitro* drug release studies were carried out at simulated intestinal fluids, and the release showed a non-Fickian anomalous transport mechanism. The drug release was found to reveal zero order kinetics. The granules and the tablets were tested for their normal physical, morphological, and analytical parameters and were found to be within the satisfactory levels. There were no significant drug-polymer interactions as revealed by infrared spectra. It has been found out that on an optimum increased Hydroxypropyl methylcellulose K100M concentration and decreased Hydroxypropyl methylcellulose K4M concentration the formulations were elegant in terms of their release profiles and were found to be statistically significant and generable.

## 1. Introduction

Diabetes is one of the major causes of death and disability in the world. World Health Organization (WHO) estimate for the number of people with diabetes worldwide, in 2000, is 171 million, which is likely to be at least 366 million by 2030. Metformin hydrochloride (Met-HCl) is a biguanide derivative of highly water soluble oral antihyperglycaemic agent used in the treatment of type II noninsulin-dependent diabetic mellitus (NIDDM) [1–3]. Some high incidence of concomitant GI symptoms, such as abdominal discomfort, nausea, and diarrhea, may occur during the treatment [4]. Gastrointestinal absorption of Met-HCl is incomplete with an absolute bioavailability of 40–60% (under fasting conditions) and in combination with rapid elimination, and 20–30% of an oral dose is recovered in faeces [2, 5]. It decreases as the dose increases, suggesting some form of saturable absorption or permeability/transit time-limited absorption [5–7]. Administration of sustained release Met-HCl form could reduce the dosing frequency and improve patient compliance. In order to achieve an optimal therapy, the effort mainly focuses on formulation of a sustained release matrix tablet of Met-HCl dosage forms [4, 8, 9].

In recent years considerable attention has been paid on the development of sustained release drug delivery systems. The piece of work employs two hydrophilic polymers Hydroxypropyl methylcellulose K4M (HPMC K4M) and Hydroxypropyl methylcellulose K100M (HPMC K100M) for the purpose of sustaining the release of Met-HCl from the dosage form for further absorption [9–12].

Our main aim of this work is to develop a formulation on two variable polymer ratios HPMC K4M and HPMC K100M for the purpose of matrixing Met-HCl in the form of tablets by the process of wet granulation [13]. Further evaluation of the tablets and granules was undertaken. The parameters included angle of repose, tapped density, bulk density, Hausner's ratio, Carr's or compressibility index for the prepared granules as preformulatory parameters. The prepared tablets were evaluated for weight variation, hardness, thickness, friability, disintegration time, and drug content [10, 14]. The dissolution of the matrix tablets was performed in simulated intestinal fluids (SIFs). The *in vitro* release mechanism of the prepared formulation was undertaken, and depending on the best zero-order release patterns of a selected formulation a 3-dimensional response surface methodology was undertaken for further data generation [12, 15].

## 2. Materials and Methods

### 2.1. Materials

Metformin hydrochloride was obtained as gift sample from Intas Pharmaceuticals, Bagheykhola, Sikkim, India. HPMC K4M and HPMC K100M were purchased from the Dow Chemical Company, USA. Isopropyl alcohol was obtained as a gift sample from Corel Pharmachem, Ahmedabad. Talc and Magnesium stearate were purchased from LOBA Chemie, Mumbai. Povidone K30 was obtained as a purchase from project funding of A.I.C.T.E-R.P.S. Microcrystalline cellulose (MCC) was purchased from Gujrat microwax Pvt. Ltd. India. All the chemicals and solvents were of analytical grade.

### 2.2. Preparation of Metformin Hydrochloride Matrix Tablets

The tablets of Met-HCl were prepared by wet granulation method by using HPMC K4M and HPMC K100M as the matrix-forming polymers, MCC was used as a diluent, Povidone K30 was used as a binder, Magnesium stearate and talc were used as lubricants. Each formulation was composed of drug and excipients in various proportions as shown in Table 1. For the formulation of tablets all the ingredients were passed through sieve no. 20 and were collected in an octagonal blender (Universal gear, Shakti Pharmaceuticals, India), mixed well to get a uniform mixture [8, 16, 17]. The paste of Povidone K30 in isopropyl alcohol was used as a granulating agent. The prepared granules were dried in an electrical drier (181824, Lab instruments and chem. works, Kolkata) at 45°C, and dried granules were further passed through sieve no. 30. Magnesium stearate and talc were added as a lubricant, and the granules were composed into tablets using a 30-station rotary punching machine (Rimek mini press 1, Shakti engineering, India).

### 2.3. Preparation of Simulated Intestinal Fluid (SIF)

1 litre of Phosphate buffer pH 6.8 (PB-6.8) was used as the primary solvent. Male Wister rats are sacrificed, and the rat caecum was collected. The fresh caecum was transversely sectioned, and the caecum contents were added to the PB-6.8. The contents were mixed properly with a stirrer (Remi motors, India). Finally the beaker containing PB-6.8 along with the rat caecal contents was incubated (Spac n service, Kolkata, India) at a temperature of 27 ± 2°C for 2 days. The final solution was filtered; pH was checked and was used for the purpose of dissolution as SIF [18].

### 2.4. Characterization of Granules

Prior to compression, the granules were evaluated for their physical parameters, such as angle of repose, bulk density, tapped density, Carr's or compressibility index [9, 10, 13, 19], Hausner's ratio as shown in Table 2.

### 2.5. Evaluation of Tablets

Tablets were evaluated for their characteristic parameters, such as weight variation, hardness, friability, thickness, disintegration time, drug content, *in vitro* dissolution study [8, 10, 11, 20] shown in Table 3.

### 2.6. Drug Polymer Interaction Study

#### 2.6.1. Fourier Transform Infrared Radiation Measurement (FT-IR)

**Figure 1:**
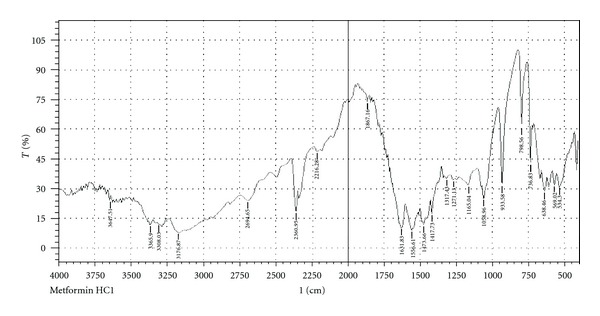
FTIR spectra of pure drug (Metformin HCl).

**Figure 2:**
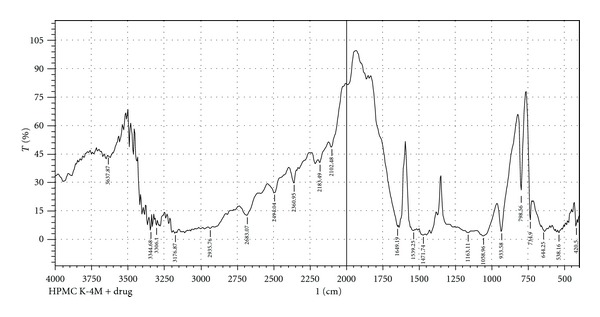
FTIR spectra of Met-HCl + HPMC K4M.

**Figure 3:**
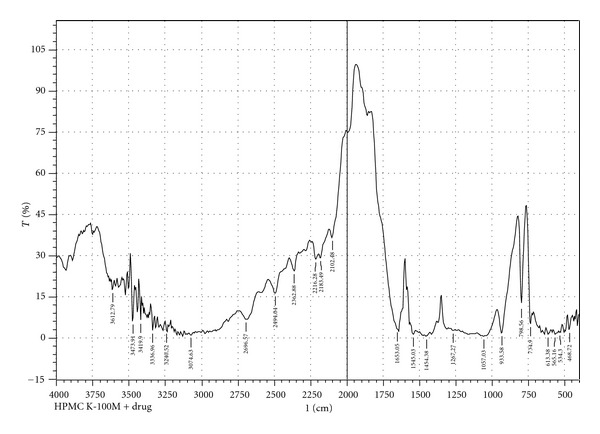
FTIR spectra of Met-HCl + HPMC K100M.

Infrared spectra of the pure drug and pure drug with several polymers were recorded on an FT-IR spectrophotometer (Model-FT-IR 8400S, Shimadzu Japan). The disc method was employed to study the possible interactions between the drug and the selected polymers. KBr (IR grade) discs in a proportion of 1 : 100: sample : KBr were prepared from the samples and eventually analyzed over arrangement of 400–4000 cm^−1^. Transmittance (T) spectra were recorded and displayed accordingly [12, 21] in Figure 1, (pure Met-HCl), Figure 2, (Met-HCl + HPMC K4M) and Figure 3, (Met-HCl+HPMC K100M).

### 2.7. Scanning Electron Microscopy (SEM)

**Figure 4:**
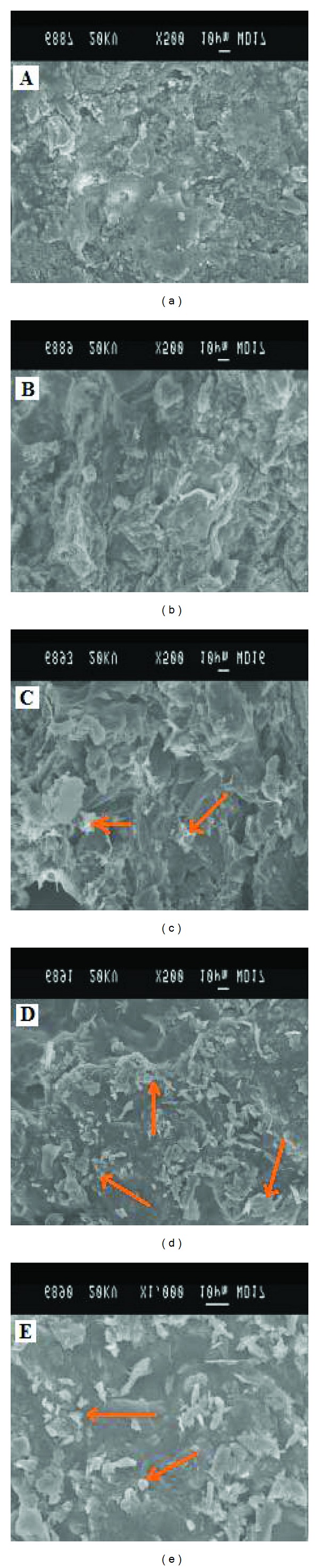
Scanning electron (SEM) photomicrographs of matrix tablets employing HPMC K4M and HPMC K100M (FF3) showing surface morphology after 0 mins ((a), 200×), 2 hrs ((b), 200×), 6 hrs ((c), 200×), 9 hrs ((d), 200×), and 9 hrs ((e), 1000×) of dissolution study. (c, d, e) arrows indicate the formation of pores in the matrices and subsequent eruption and leaching of drug.

Tablet samples (FF3) were removed from dissolution apparatus at predetermined time intervals (0 min, 2 hours, 6 hours, 9 hours) and the upper surface of the tablet was scrapped off by sectioning transversally from the concave face of the tablet. The specimen was then placed on a sample holder so as to present surface and cross-sectional view of the tablet to the microscope. Samples were coated with gold and visualized under scanning electron microscope (SEM) [8, 12] JEOL, JSM 840A, Japan (Figure 4).

### 2.8. *In Vitro* Release Studies

*In vitro* drug release from tablets was studied using a USP-II type dissolution apparatus (paddle type) in TDT 08L model (Electrolab, Kolkata). The study was carried out in 900 mL of SIF at 37 ± 0.5°C. Sink condition was maintained for the whole experiment. Ten milliliters of the sample was withdrawn at regular intervals, and the same volume of warmed (37 ± 0.5°C) fresh dissolution medium was replaced to maintain the volume constant. The samples withdrawn were filtered, and the drug content in each sample was analyzed after being suitable with a Shimadzu UV-1700 Pharma Spectroscopy, Japan at 233 nm [8, 9, 13, 17].

### 2.9. *In Vitro* Drug Release Kinetic Study

The dissolution data were subjected to release kinetic study. Drug dissolution from solid dosage form has been described by kinetic models in which the dissolved amount of drug (*Q*) is compared to the function of the test time (*t*). Some analytical definitions and kinetic models of the *Q* versus *t* commonly used are Zero order, First order, Weibull, Higuchi, and Korsmeyer-Peppas.

#### 2.9.1. Zero Order Release Kinetics [8]

**Figure 5:**
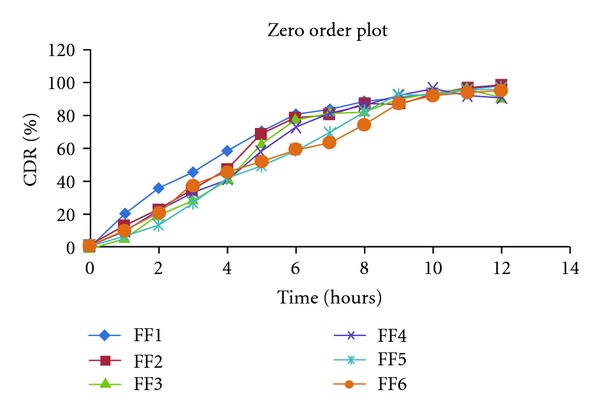
Zero order kinetics data of formulations. %CDR percentage of cumulative drug release.

It defines a linear relationship between the fractions of drug release versus time:

(1) $$\begin{array}{c} Q=K_{0}t, \end{array}$$

where *Q* is the fraction of drug release at time *t* and *K*_0_ is the zero order release rate constant. A plot of fraction of drug release against time will be linear, if the release obeys zero order release kinetics shown at Table 4 and Figure 5.

#### 2.9.2. First Order Release Kinetics [8, 10]

**Figure 6:**
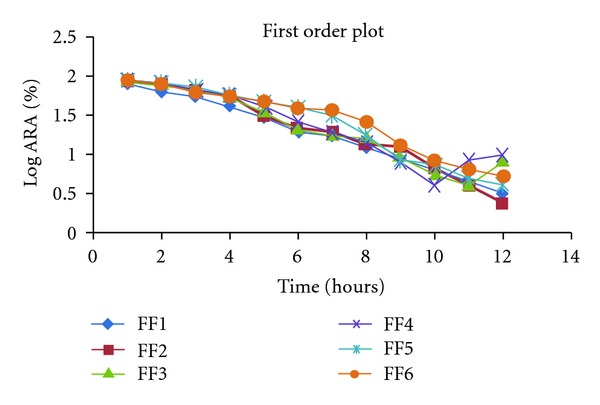
First order release kinetics data of formulations.

 Assuming that the exposed surface area of a tablet decreases exponentially with time during dissolution process, suggest that drug release from most of the slow release tablets could be described adequately by apparent first order kinetics. The equation that describes first order kinetic is

(2) $$\begin{array}{c} \mathrm{In}\left(1-Q\right)=-K_{1}t, \end{array}$$

where *Q* is the fraction of drug released at time *t* and *K*_1_ is the first order release rate constant. A plot of the logarithm of the fraction of the drug remaining against time will be linear if the release obeys first order release kinetics shown at Table 4 and Figure 6.

#### 2.9.3. Higuchi Kinetic Model [8, 10]

**Figure 7:**
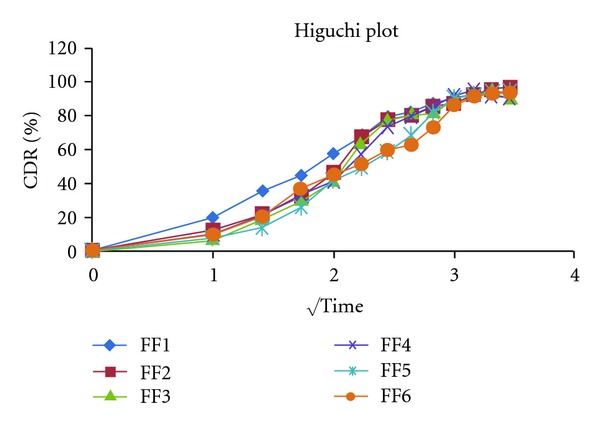
Higuchi release kinetics data of formulations.

 It defines a linear dependence of the active fraction released per unit of square root of time:

(3) $$\begin{array}{c} Q=K_{2}t^{1/2}, \end{array}$$

where *K*_2_ is release rate constant. A plot of the fraction of drug released against square root of time will be linear if the release obeys Higuchi equation shown at Table 4 and Figure 7.

#### 2.9.4. Power Law (Korsmeyer and Peppas Equation) [8, 10]

**Figure 8:**
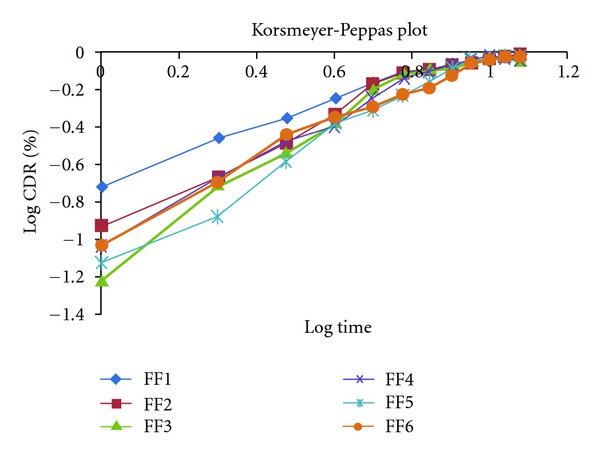
Korsmeyer-Peppas kinetics graph for all formulations

 In order to define a model, which would represent a better value for the dissolution data, it was further analyzed by Korsmeyer Peppas and equation:

(4) $$\begin{array}{c} \text{Log}\frac{Mt}{M\infty }=n\;\text{Log}\;t+\text{Log}\;k, \end{array}$$

where *Mt* is the amount of drug released at time *t* and *Mα* is the amount released at time *α*; thus the *Mt*/*Mα* is the fraction of drug released at time *t*, *K* is the kinetic constant, and *n* is the diffusion exponent. A plot between log of *Mt*/*Mα* against log of time will be linear if the release obeys Korsmeyer-Peppas equation and the slope of this plot represents “*n*” value. This enables the interpretation of diffusion exponent and solute release mechanism for cylindrical shape release mechanism from polymeric film shown at Table 5 and Figure 8.

**Weibull Equation**

Consider

(5) $$\begin{array}{c} \text{Log}\left\{-\ln\left(1-\frac{Mt}{M\infty }\right)\right\}=b\;\log t+\log t_{d}. \end{array}$$

The *b*-value obtained from the Weibull equation (V) has certain criteria such as the following.

- *b* < 0.35 not found in simulation studies may occur in highly disordered spaces that must be different from the percolating cluster.
- *b* ~ 0.35–0.39: diffusion fractal substrate morphologically similar to the percolated cluster.
- 0.39 < *b* < 0.69: diffusion in fractal or disorder substrate different from the percolation cluster. These values were not observed in Monte Carlo simulation results. It is however plausible to assume this possibility since there has to be a crossover from fractal to euclidian dimension.
- *b* ~ 0.69–0.75: diffusion in normal euclidean space.
- 0.75 < *b* < 1: diffusion in normal euclidean substrate with contribution of another release mechanism. In this case, the power law can describe the entire set of data of a combined release mechanism.
- *b* = 1: first order release obeying Fick's law of diffusion, the rate constant which controls the release kinetics.
- *b* > 1: Sigmoid indicative of complex release mechanism; the rate of release increases up to the inflection point and thereafter declines [8, 10, 22].

**Figure 9:**
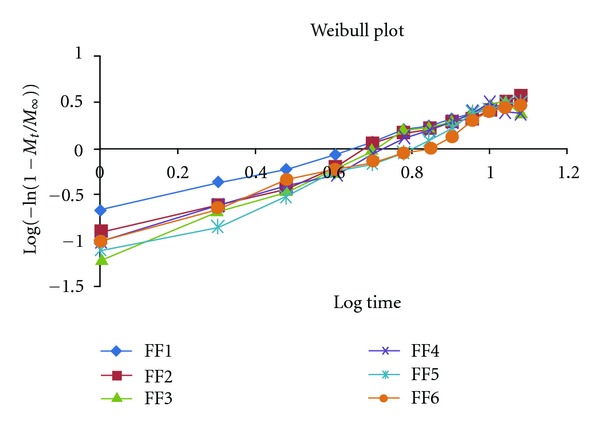
Weibull function (*b* value) kinetics graph for all formulations.

The values are shown at Table 5 and Figure 9.

## 3. Statistical Optimization

**Figure 10:**
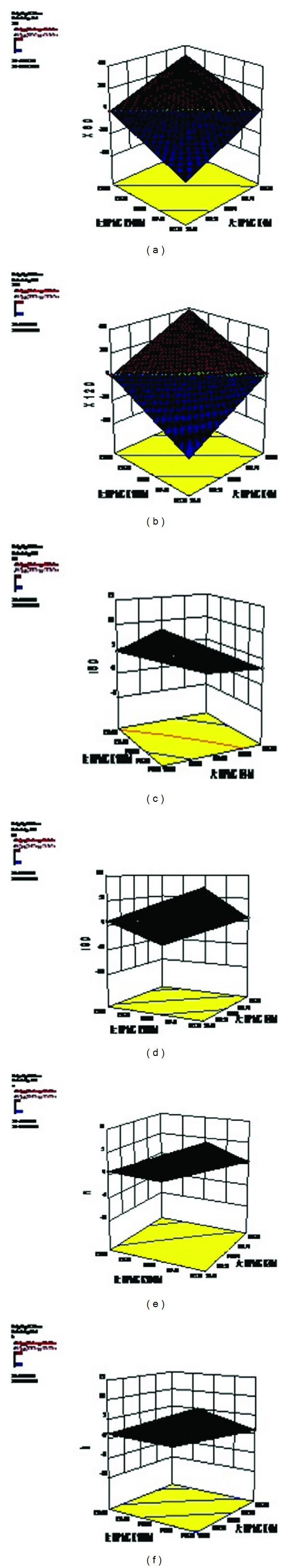
3D response surface curves for the effect of selected independent variables (*X*1 and *X*2) on dependent responses *X*_60_, *X*_120_, *T*_50_, *T*_90_, *n*, and *b* values, respectively.

Statistical optimization was done with the help of Design-Expert 8.0.4 software, USA with the help of the data obtained from the dissolution of a selected formulation FF3 keeping two different ratios of polymers in two axes as the independent variables and *X*_60_ (amount released after 60 minutes), *X*_120_ (amount released after 120 minutes), *T*_50_ (time taken for the formulation to release 50% of the active medicament), *T*_90_ (time taken for the formulation to release 90% of the active medicament), “*n*” (*n*-values obtained from Korsmeyer-Peppas), or “*b*” (*b*-value obtained from weibull) value on the third axis as fixed parameters [8, 22, 23]. The comparisons of the values are shown in Table 6. The 3-dimensional response surface was shown in Figure 10.

## 4. Result and Discussion

In the preformulation study, prepared granules were evaluated for various physical properties. The granules indicated good or passable flowability with an angle of repose values ranging from 29 to 32°. The bulk densities for the granules ranged between 0.482 ± 0.06 and 0.593 ± 0.02 g/cm^3^. The tapped densities for the granules ranged between 0.573 ± 0.02 and 0.701 ± 0.06 g/cm^3^. This value of bulk and tapped density indicates good packing character. The compressibility index (Carr's index) for all the formulations was found to be almost below 18%, which indicates desirable good flow properties. Hausner's ratio was also calculated for the granules flow property determination and seems to be within the range, that is, 1.15 to 1.23.

An evaluation of physicochemical testing results of all the formulations showed acceptable range. The weight variation indicated that all the tablets were uniform with low standard deviation values. The tablets mean thickness values range from 5.99 ± 0.08 to 6.76 ± 0.05 mm. The hardness of all the tablets was within the range of 6.1 ± 0.19 to 7.9 ± 0.16 kg/cm^2^. The loss in total weight in friability test was in range of 0.21 to 0.78%. The percentage drug content for different tablets formulation varied from 498.4 ± 1.12 to 499.7 ± 0.56 and was found to be within limits, which indicates uniform drug distribution in all the formulations

The final set of the formulations FF1 to FF6 was subjected to dissolution study at SIF; the release profile of formulation was fitted to Zero order, First order, Higuchi, Korsmeyer-Peppas, and Weibull kinetic model. Percentage cumulative drug release (%CDR) of the formulation FF1 to FF9 in 10 h was found to be in the range 91.64 to 95.98. On comparing the *R* ^2^ values of the formulations it was observed that all the formulations follow zero order kinetics as the values were nearer to the unity. In order to verify the release pattern the Korsmeyer-Peppas and Weibull kinetics had been employed. According to the “*n*” values of Korsmeyer-Peppas model almost all the formulations showed to follow non-Fickian anomalous law of diffusion. Further intrinsic study was performed considering variation in the shape factor “*b*.” Therefore Weibull function was determined for each formulation, which reveals a new pattern regarding the release of the drug. The “*b*” value of all the formulations comes above “1” which suggests that the formulations follow a sigmoidal curve indicative of complex release mechanism, the rate of release increases up to the inflection point and there after declines. Formulation FF3 containing HPMC K4M (102 mg) and HPMC K100M (148.7 mg) showed elegant dissolution parameters (*X*_60_ = 6.15%, *X*_120_ =19.80%, *T*_50_ = 4.22 h, *T*_90_ = 8.56 h, *n* = 1.085, *b* = 1.587). The mathematical relationship was constructed for the studied response variables. Final equation in terms of actual factors: *X*_60_ = 9.59 + 246.82 ∗ *A* + 241.62 ∗ *B* − 229.88 ∗ *A* ∗ *B* − 225.28 ∗ *A* ^2^, *X*_120_ = 20.45 + 192.67 ∗ *A* + 184.43 ∗ *B* − 6.30 ∗ *A* ∗ *B*, *t*_50_ = 4.46 − 4.17 ∗ *A* − 3.50 ∗ *B* + 0.53 ∗ *A* ∗ *B*, *t*_90_ = 8.07 − 37.57 ∗ *A* − 37.13 ∗ *B* − 0.84 ∗ *A* ∗ *B*, *n* = 0.99 − 4.32 ∗ *A* − 4.16 ∗ *B* + 0.20 ∗ *A* ∗ *B* and *b* = 1.50 − 4.49 ∗ *A* − 4.36 ∗ *B* + 0.24 ∗ *A* ∗ *B*. It was observed that basic physicochemical properties as well as dissolution profile of all the tablet formulations entirely depend on the combination of polymers incorporated in it. FF3 showed elegant and satisfactory result in terms of physicochemical parameters and release kinetics; the polymers concentration was considered optimum with HPMC K4M (102 mg) and HPMC K100M (148.7 mg), HPMC being both hydrophilic and lipophilic in nature. Basically the polymer composition was found to have a direct influence on dissolution profile. As revealed from the kinetics it was found out that there was an initial burst effect of the formulations, followed by sustained release to provide maintenance dose of the drug.

During preformulation study, FTIR (Fourier transform infrared) spectroscopy study of the pure drug Met-HCl alone and in combination with polymers (HPMC K4M, HPMC K100M) under study was observed. IR spectra are given in Figures 1, 2, and 3 shows C–H alkane stretch at values of 2950 (pure Met-HCl), 2985.76 (Met-HCl + HPMC K4M), 3010.36 ((Met-HCl + HPMC K100M). –NH at 3365.90 (pure Met-HCl), 33.44.68 (Met-HCl + HPMC K4M), 32.40.52 ((Met-HCl + HPMC K100M). For –C=N the values were 1473.66 (pure Met-HCl), 1471.74 (Met-HCl + HPMC K4M), 1454.38 (Met-HCl + HPMC K100M). Major frequencies of functional groups of pure drug remained unchanged in presence of polymers. Hence chances of less possible major interaction between the drug and the polymers can be concluded.

The SEM topography of dry tablet (Figure 4) shows its surface nonuniformity with membrane structure of the pores and drug particles whereas wet and dry tablet surface shows drug and excipients particles along with the membrane like gel structure. The images of the dry tablet surface showed a degree of mechanical interlocking of the tablet excipients particles. Structure of sectioned tablet before being wet shows nonuniformity of gel structure with pores and structure of tablet after wetting shows well-formed gel with less number of pores by the polymer relaxation upon absorption of water.

## 5. Conclusion

From the previous piece of work it is obvious that Met-HCl tablets can be generated from a lab-scale to a fully feasible industrial scale with no/less drawbacks with better optimization procedures. The data obtained from this piece of work can definitely open newer dimension for further research on matrix tablets.

## Figures and Tables

**Table 1:** Formulation design of Met-HCl matrix tablets.

Sl. no.	Ingredients (mg)	Formulation					
		FF1	FF2	FF3	FF4	FF5	FF6
1	Metformin HCl	500	500	500	500	500	500
2	Microcrystalline cellulose	101	100.2	99.3	98.4	97.5	96.6
3	HPMC K4M (X1)	103.8	102.9	102	101.2	100.3	99.4
4	HPMC K100M (X2)	145.2	146.9	148.7	150.4	152.2	154
5	Povidone K30	20	20	20	20	20	20
6	Magnesium stearate	5	5	5	5	5	5
7	Talc	5	5	5	5	5	5

**Table 2:** Evaluation of granules.

Sl. no.	Batch code	Evaluating parameters				
		Angle of repose	Bulk density	Tapped density	Carr's index	Hausner's ratio
1	FF1	31.23 ± 0.07	0.593 ± 0.02	0.701 ± 0.06	15.41 ± 0.08	1.18
2	FF2	30.89 ± 0.08	0.561 ± 0.07	0.673 ± 0.03	16.64 ± 0.11	1.2
3	FF3	29.16 ± 0.11	0.497 ± 0.04	0.607 ± 0.07	18.12 ± 0.07	1.22
4	FF4	30.72 ± 0.06	0.482 ± 0.06	0.573 ± 0.02	15.73 ± 0.09	1.19
5	FF5	31.52 ± 0.13	0.532 ± 0.09	0.613 ± 0.08	13.21 ± 0.14	1.15
6	FF6	32.69 ± 0.14	0.513 ± 0.08	0.629 ± 0.05	18.44 ± 0.13	1.23

All values represent mean ± SD (*n* = 3).

**Table 3:** Evaluation of tablets.

		Evaluating parameters					
Sl. no.	Batch code	Wt. variation (mg)	Hardness (kg/cm^2^)	Thickness (mm)	Friability (%)	Disintegration time (hr. min.)	Drug content (%)
1	FF1	878.87 ± 2.35	6.6 ± 0.16	6.29 ± 0.08	0.59	3.17	499 ± 0.84
2	FF2	881.41 ± 2.21	6.3 ± 0.14	6.76 ± 0.05	0.46	3.03	499.4 ± 0.31
3	FF3	880.12 ± 1.91	7.8 ± 0.21	6.42 ± 0.07	0.63	2.44	499.7 ± 0.56
4	FF4	878.86 ± 3.16	6.1 ± 0.19	6.36 ± 0.03	0.39	2.5	498.9 ± 1.21
5	FF5	879.23 ± 2.86	7.6 ± 0.23	6.01 ± 0.09	0.78	2.37	499.2 ± 0.67
6	FF6	882.36 ± 1.78	7.9 ± 0.16	5.99 ± 0.08	0.21	2.52	498.4 ± 1.12

All values represent mean ± SD (*n* = 6).

**Table 4:** Values obtained after fitting the drug release data of all formulations to different kinetic model.

Batch code	Zero order		First order		Higuchi model	
	*R* ^2^	*K*_0_	*R* ^2^	*K*_1_	*R* ^2^	kH
FF1	0.894	7.741	0.994	−0.13	0.975	0.031
FF2	0.918	8.508	0.972	−0.14	0.944	0.028
FF3	0.907	8.673	0.933	−0.124	0.924	0.027
FF4	0.915	8.471	0.882	−0.118	0.931	0.028
FF5	0.967	8.944	0.953	−0.135	0.928	0.027
FF6	0.972	8.202	0.94	−0.117	0.957	0.03

**Table 5:** *n*-values and *b*-values obtained according to Korsmeyer-Peppas and Weibull kinetic model.

Formulations	“*n*” values	Type of transport	“*b*” values	Type of transport
FF1	0.651	Non-Fickian	1.145	Sigmoid indicative of complex release mechanism
FF2	0.889	Non-Fickian anomalous	1.415	Sigmoid indicative of complex release mechanism
FF3	1.085	Non-Fickian anomalous	1.587	Sigmoid indicative of complex release mechanism
FF4	0.951	Non-Fickian anomalous	1.435	Sigmoid indicative of complex release mechanism
FF5	1.1	Non-Fickian anomalous	1.612	Sigmoid indicative of complex release mechanism
FF6	0.921	Non-Fickian anomalous	1.355	Sigmoid indicative of complex release mechanism

**Table 6:** Comparison of *X*_60_, *X*_120_, *T*_50_, *T*_90_, *n*, and *b* value of formulations.

Batch code	*X*_60_ (mg)	*X*_120_ (mg)	*T*_50_ (hr.)	*T*_90_ (hr.)	*n*	*b*
AFF1	13.35	35.29	3.23	8.35	0.651	1.145
AFF2	11.87	21.6	4.13	9.26	0.889	1.415
AFF3	6.15	19.8	4.22	8.56	1.085	1.587
AFF4	9.24	21.61	4.34	8.36	0.951	1.435
AFF5	7.47	13.23	5.1	8.51	1.1	1.612
AFF6	9.25	20.31	4.44	9.4	0.921	1.355
